# Association of Children’s Hospital Status With Value for Common Surgical Conditions

**DOI:** 10.1001/jamanetworkopen.2022.18348

**Published:** 2022-06-24

**Authors:** Mehul V. Raval, Audra J. Reiter, Ian M. McCarthy

**Affiliations:** 1 Department of Surgery and Pediatrics, Surgical Outcomes and Quality Improvement Center, Center for Healthcare Studies, Institute of Public Health and Medicine, Northwestern University Feinberg School of Medicine, Chicago, Illinois; 2 Division of Pediatric Surgery, Department of Surgery, Ann & Robert H. Lurie Children’s Hospital of Chicago, Chicago, Illinois; 3 Department of Economics, Emory University, Atlanta, Georgia

## Abstract

**Importance:**

Although children’s hospitals (CH) provide a substantial proportion of highly specialized pediatric care in the United States, the value of CH compared with non–children’s hospitals (NCH) for routine surgical procedures is unknown.

**Objective:**

To examine the value of CH for routine surgical procedures by assessing clinical outcomes and payment data.

**Design, Setting, and Participants:**

This retrospective cohort study examined pediatric patients undergoing 1 of 13 commonly performed surgical procedures between 2010 and 2015 with 90-day follow-up using administrative data from the Health Care Cost Institute. Data analysis was conducted from July 2019 to December 2021.

**Exposures:**

The primary exposure was tier of CH status, defined using self-reported pediatric services, affiliation with pediatric focused programs, and validated based on proportion of pediatric admissions.

**Main Outcomes and Measures:**

Payments for common surgical procedures from private insurers and overall complication and readmission rates at 30, 60, and 90 days.

**Results:**

There were 368 220 pediatric patients who underwent one of the surgical procedures of interest; 220 899 (60.0%) of the patients were male; 118 977 (32.3%) had their procedure at freestanding CH (CH-A), 75 256 (20.4%) at CH attached to an adult hospital (CH-B), and 173 987 (47.3%) at NCH. The mean (SD) payment for all procedures at CH-A was $6533.56 ($6399.97), $5847.50 ($4947.47) at CH-B, and $5034.25 ($4787.07) at NCH. The mean (SD) overall complication rate was 0.004 (0.06) at CH-A, 0.01 (0.07) at CH-B, and 0.003 (0.06) at NCH. Readmission rates at 30, 60, and 90 days were similar across all hospital types. After adjusting for zip code, year, surgery, surgery setting, and observable patient, hospital, and county characteristics, the estimated payments for inpatient common procedures were 39% higher at CH-A than at NCH. Payments for outpatient common procedures were 34% higher at CH-A than at NCH.

**Conclusions and Relevance:**

In this cohort study, children who underwent common surgical procedures had equivalent clinical outcomes at CH and NCH but the procedures were associated with higher payments and, thus, overall lower value care. To ensure delivery of optimal value to patients and payers, more research is needed to evaluate mechanisms to ensure access, decrease costs, and improve value at both CH and NCH.

## Key Points

**Question:**

Do children’s hospitals provide higher value care for routine surgical procedures compared with non–children’s hospitals?

**Findings:**

In this cohort study of 368 220 pediatric patients, negotiated payments for commonly performed surgical procedures in children were significantly higher at children’s hospitals than non–children’s hospitals. Inpatient procedures were 39% higher and outpatient procedures were 34% higher.

**Meaning:**

This study’s findings suggest that for routine surgical procedures, children’s hospitals have comparable clinical outcomes but higher payments, and thus lower value, compared with non–children’s hospitals.

## Teaser

This cohort study examines the value of children’s hospitals vs non–children’s hospitals for routine surgical procedures for children by assessing clinical outcomes and payment data.

## Introduction

Although children’s hospitals (CH) comprise less than 5% of hospitals in the United States, CH account for 40% of pediatric inpatient days and 50% of costs for pediatric care.^1^ CH often provide high-volume, specialized, and resource-intensive care to children who require highly trained clinicians and innovative technologies. One example is congenital heart surgery where mortality rates are lowest at high-volume, specialized centers.^2,3^ For this type of highly specialized pediatric care, the value proposition of higher costs at CH is arguably justified by demonstrable improved outcomes and quality.^4^ In 2009, 40 freestanding CH accounted for greater than $10 billion of annual US health care expenditure, and the top 10 CH profited more than $800 million.^5^ Contemporary pediatric care has witnessed substantial regionalization in the last decade, and there are efforts underway to centralize the delivery of children’s surgical care to specialized centers.^6,7,8,9^

Although CH have been shown to provide higher quality care than non–children’s hospitals (NCH) for highly specialized procedures, there are data to suggest the cost of common and routine procedures is also greater at CH than NCH.^10,11^ Despite surgical interventions representing high-risk and costly experiences in our health care system, little attention has been directed at surgeons, surgical care or surgical payment reform, transparency of surgical outcomes, and consumer/patient empowerment in choices surrounding surgical care.^12,13^ Of the 50 most prevalent and costly pediatric inpatient conditions, 32 are surgical.^14^ Furthermore, surgical care accounts for a high proportion of overall health care spending.^15^ The financial and clinical implications of trends and policies related to children’s surgical care have not been fully evaluated and may result in a substantial rise in health care costs without demonstrable improvement in quality.^6,16^

The primary objective of this study was to determine the value of CH for routine surgical procedures by assessing clinical outcomes and payments data. We compared quality by assessing complication and readmission rates, and price, using payment data of commonly performed surgeries at CH and NCH. We then explored the extent to which quality and price differences could be explained by patient and hospital characteristics vs other economic factors such as hospital and insurer market structure.

## Methods

### Study Design and Data Source

This was a retrospective cohort study using version one of the Health Care Cost Institute (HCCI) data set. The HCCI provides deidentified administrative cost and utilization data for over 10 million beneficiaries in the United States covered by private insurance and is ideal for evaluating variation in hospital-level pricing and payments. These data consist of claims submitted to HCCI by Aetna, Humana, Kaiser Permanente, and UnitedHealthcare.^17^ We focus on the pediatric population, where the HCCI data purportedly cover approximately 25% of all claims for privately insured children in the US.^18^ HCCI data have previously been used to evaluate variations in prices between states and Metropolitan Statistical Areas (MSA).^19^ The Ann and Robert H. Lurie Children’s Hospital of Chicago’s institutional review board deemed this study exempt from review and a waiver of informed consent was granted because the study was determined to be minimal risk and because data are deidentified. This study followed the Strengthening the Reporting of Observational Studies in Epidemiology (STROBE) reporting guideline for cohort studies.^20^

### Study Cohort

We analyzed claims data from January 2010 to September 2015 from HCCI.^1719^ From this population of privately insured beneficiaries, we included patients aged 18 years or younger, who underwent commonly performed pediatric surgical procedures. We examined outcomes and costs following 13 procedures: anterior cruciate ligament (ACL) reconstruction, antireflux surgery, appendectomy, humerus fracture repair, tympanostomy tube placement, tonsillectomy and adenoidectomy, strabismus surgery, posterior spinal fusion, cholecystectomy, umbilical hernia repair, inguinal hernia repair, orchiopexy, and circumcision. Patients who underwent multiple procedures at the same visit (eg, both tonsillectomy and tympanostomy) were classified as concurrent procedures. Final procedure inclusion was determined through a combination of literature review and clinical judgement to purposefully capture inpatient and outpatient populations and to represent the full spectrum of children’s surgical procedures performed at most hospitals.^14^ Procedures were identified with *Current Procedural Terminology (CPT)* and *International Classification of Diseases, Ninth Revision (ICD-9)* procedure codes using facility and professional claims (eTable 1 in the Supplement). We excluded newborns, patients who were transferred, and outliers, defined as payments below the 5th or above the 95th percentile of payment ratios.

### Hospital Classification

CH were distinguished from NCH using a previously described method.^21^ In brief, hospitals were categorized using a combination of self-reported pediatric services on the American Hospital Association (AHA) Survey followed by validation using publicly available data on hospital membership in various pediatric programs such as the Children’s Hospital Association, Children’s Oncology Group, and American College of Surgeons National Surgical Quality Improvement Program–Pediatric. Using this methodology, 3 tiers of hospitals were created. A final validation used HCCI claims data to determine the proportion of pediatric admissions at each hospital. Furthermore, hospitals were classified as teaching or nonteaching if they reported being a member of the Council of Teaching Hospital of the Association of American Medical Colleges on the AHA survey.

### Outcome Measures

Postoperative outcomes were identified using *ICD-9* codes using facility and professional claims for wound complications, surgical site infections, urinary tract infections, renal insufficiency, pneumonia, respiratory failure, sepsis, deep vein thromboses, pulmonary embolism, cardiac complications, intraoperative complications, and 30-day, 60-day, and 90-day readmissions (eTable 2 in the Supplement). These occurrences include emergency department claims that were part of a hospital facility, critical access hospital, or surgery center. Negotiated payment rates and patient characteristics for each procedure were obtained from the HCCI database.

### Statistical Analysis

We tested for differences in unadjusted mean payments and quality outcomes by hospital type. For complications, we used the χ^2^ test; and for prices, we used the *t* test and nonparametric Kruskal-Wallis test. For all statistical tests, the significance was set at *P* < .05 and all testing was 2-sided

Then we examined differences in payments and quality with regressions, where we controlled for observable patient variables (gender and comorbidities), the presence of complex chronic conditions, procedure type, and hospital characteristics. Complex chronic conditions were accounted for using methodologies outlined by Feudtner et al.^22,23,24,25^ We used regression models with market, year, and procedure fixed effects to examine differences in mean prices and complication rates conditional on covariates. More formally, we estimated by ordinary least squares (OLS) using the following regression model:


*Y_i(hmt)t_ = βX_it_ + γZ_ht_ + λW_mt_ + δCH_i_ + θ_g_ + θ_t_ + θε_it_*

*Y_i(hmt)_* denotes the outcome (eg, log price) for patient *i* with insurance product *g* at hospital *h*, market *m*, and year and month *t*; *X_it_* denotes patient and procedure characteristics including an indicator for the procedure, whether the patient has any complex chronic conditions, the inpatient vs outpatient setting, and whether the patient is female; *Z_ht_* denotes hospital characteristics from the AHA survey data, including bed size, number of nurses, physicians, residents, and other full-time equivalents, total hospital discharges, total Medicare discharges, and total Medicaid discharges; *W_mt_* denotes market-level variables from the American Community Survey, including percentage of residents of different age categories, race, income, and education; CH denotes an indicator for whether the hospital is a children's hospital; *θ_g_* and *θ_t_* capture fixed effects for the patient's insurance product (the insurance group ID) and year and month fixed effects; and *ε_it_* is an error term. Standard errors are robust to heteroskedasticity and clustering at the hospital level. In cases where the outcomes were binary, such as for 90-day readmissions or complications, we estimated the same specification using a generalized linear model with a binomial family and a logit link function.

The regression specification included a set of indicator variables for the care setting (inpatient vs outpatient) and the specific procedure; however, these indicator variables likely do not fully capture important differences between CH and NCH. Therefore, in addition to an overall analysis of all procedures and all settings, we estimated this regression separately for each procedure and separately for the inpatient and outpatient settings. For prices, there are 26 regressions total, but not all results are available for these individual analyses due to small sample sizes. Similarly, for regressions involving quality outcomes, we focused on the full inpatient sample due to low counts of readmissions or complications for individual procedures or outpatient-only procedures.

All HCCI data were accessed remotely via Citrix Workspace. The claims data were stored in a Vertica database, from which an analytic data set was created and managed using SAS version 9.4 (SAS Institute). All statistical analyses were performed using Stata version 15 (StataCorp) and R (the R Project for Statistical Computing) from July 2019 to December 2021.

## Results

### Patient and Hospital Characteristics

**Figure 1.**
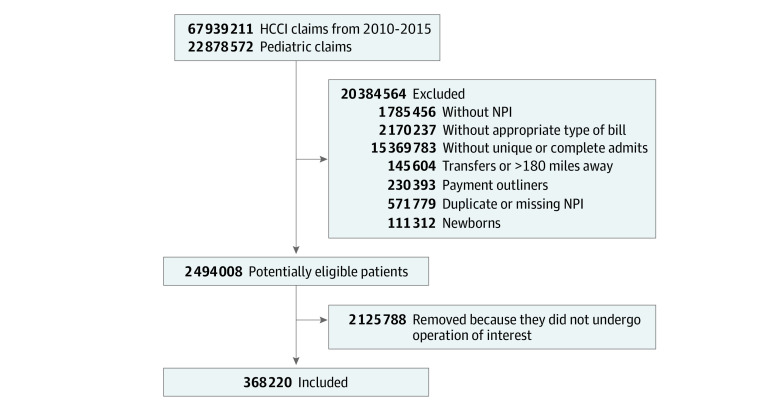
Study Flowchart HCCI indicates Health Care Cost Institute; NPI, National Provider Identifier.

Of the 67 939 211 patients represented in HCCI data spanning from January 1, 2010, to December 31, 2015, 22 878 572 (33.7%) were aged 18 years or younger. Of these patients, 368 220 (1.6%) were identified to have undergone one of the index surgical procedures of interest. This cohort of patients was assigned to CH, subdivided into 118 977 (32.3%) at freestanding children’s hospitals (CH-A), 75 256 (20.4%) at children’s hospital attached to adult hospitals (CH-B), and 173 987 (47.3%) at NCH (Figure 1).

The 368 220 patients (220 899 [60.0%] male patients) included in this analysis were seen across 12 669 hospitals; 280 hospitals (2.2%) were CH-A, 1079 hospitals (8.5%) were CH-B, and 11 310 hospitals (89.3%) were NCH; 72 576 patients (61%) were male at CH-A, 47 411 male patients (63%) at CH-B, and 100 912 patients (58%) at NCH. The mean (SD) number of beds at CH-A was 263 (122), CH-B was 647 (328), and NCH was 210 (173); 274 CH-A (98%) were nonprofit hospitals whereas 777 CH-B (72%) and 8143 NCH (72%) were nonprofit; 101 CH-A (36%), 604 CH-B (56%), and 679 NCH (6%) were teaching institutions (Table 1).

**Table 1.** Characteristics of Patients Undergoing Surgery at Children’s Hospitals and Non–Children’s Hospitals

Characteristics	Total	CH-A	CH-B	NCH
Hospitals	12 669	280	1079	11 310
Patients	368 220	118 977	75 256	173 987
Patient-level characteristics, No. (%)				
Female	147 321 (40.0)	46 401 (39.0)	27 845 (37.0)	73 075 (42.0)
Male	220 899 (60.0)	72 576 (61.0)	47 411 (63.0)	100 912 (58.0)
Hospital-level characteristics				
Bed size, mean (SD)	248 (190)	263 (122)	647 (328)	210 (173)
Nonprofit, No. (%)	9194 (72.6)	274 (97.9)	777 (72.0)	8143 (72.0)
Teaching, No. (%)	1384 (10.9)	101 (36.1)	604 (56.0)	679 (6.0)
Procedures, No.				
Strabismus surgery	13 615	6339	3232	4044
Tympanostomy tube placement	99 254	33 614	15 108	50 532
Tonsillectomy and adenoidectomy	104 163	28 640	18 302	57 221
Repair of humerus fracture	14 719	5480	3749	5490
ACL repair	736	123	107	506
Posterior spinal fusion for scoliosis	4384	2027	1282	1075
Anti-reflux surgery	876	312	387	177
Cholecystectomy	426	76	87	263
Appendectomy for acute appendicitis	35 471	8906	8303	18 262
Umbilical hernia repair	8241	3535	2682	2024
Inguinal hernia repair, non-obstructive	16 273	6503	5261	4509
Orchiopexy for undescended testicles	7831	3199	2605	2027
Circumcision	16 666	5864	4857	5945
Concurrent procedures	45 565	14 359	9294	21 912

Abbreviation: ACL, anterior cruciate ligament.

### Surgical Procedures

A variety of surgical procedures across multiple pediatric surgical subspecialties were evaluated. Tonsillectomy and adenoidectomy was the most common procedure performed with 104 163 cases; 28 640 (27.5%) were performed at CH-A, 18 302 (17.6%) at CH-B, and 57 221 (54.9%) at NCH. Cholecystectomy was the least common procedure with 426 cases; 76 (17.8%) were performed at CH-A, 87 (20.4%) at CH-B, and 263 (61.7%) at NCH. There were 45 565 patients who had concurrent procedures done under the same anesthetic, 14 359 (31.5%) at CH-A, 9294 (20.4%) at CH-B, and 21 912 (48.1%) at NCH (Table 1).

### Payments

**Figure 2.**
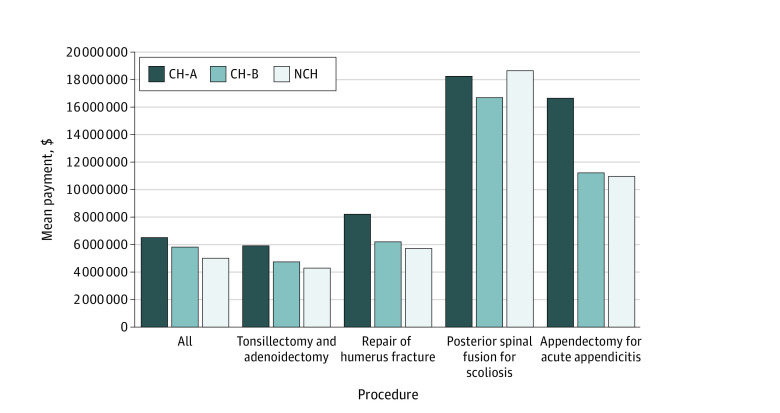
Mean Payments From Commercial Insurers Using Health Care Cost Institute Data for Common Procedures at Children’s Hospitals (CH) and Non–Children’s Hospitals (NCH) CH-A indicates CH tier A (freestanding CH); and CH-B, CH tier B (CH attached to adult hospital).

The mean (SD) payment from commercial insurers for all procedures was $6553.56 ($6399.97) at CH-A, $5847.50 ($4947.47) at CH-B, and $5034.25 ($4787.07) at NCH. Appendectomy for acute appendicitis had the largest difference in payments with CH-A receiving $5618.75 more in payment than NCH. Posterior spinal fusion was the only procedure where NCH received higher payments than CH, with NCH receiving $406.50 more than CH-A and $1947.06 more than CH-B (Figure 2).

### Complications

There was no significant difference in the rate of surgical complications or readmissions within 30, 60, or 90 days of surgery at any of the hospital types. The mean (SD) overall complication rate was 0.004 (0.06) at CH-A, 0.01 (0.07) at CH-B, and 0.003 (0.06) at NCH. Readmission rates at 30, 60, and 90 days were similar across all hospital types (Table 2). Adjusting for observable characteristics, we again see no significant difference in readmissions or complications among CH-A or CH-B compared with NCH. For example, a coefficient estimate of 0.23 (Table 3) implies an estimated 1.26 increase in the odds of a complication, or a 26% increase in complication rates, for CH-A compared with NCH. However, given the low rates of complications overall, this change in odds equates to less than a 0.15% higher complication rate for CH-A compared with NCH.

**Table 2.** Rate of Surgical Complications and Readmissions

Procedures	Rate, mean (SD)											
	Any complication			Readmissions								
				30 d			60 d			90 d		
	CH-A	CH-B	NCH	CH-A	CH-B	NCH	CH-A	CH-B	NCH	CH-A	CH-B	NCH
All procedures	<0.01 (0.06)	0.01 (0.07)	<0.01 (0.06)	0.01 (0.1)	0.01 (0.11)	0.01 (0.08)	0.01 (0.12)	0.01 (0.12)	0.01 (0.1)	0.02 (0.13)	0.02 (0.13)	0.01 (0.1)
Tonsillectomy and Adenoidectomy	<0.01 (0.04)	<0.01 (0.04)	<0.01 (0.02)	0.01 (0.11)	0.01 (0.11)	0.01 (0.08)	0.01 (0.12)	0.01 (0.12)	0.01 (0.08)	0.02 (0.12)	0.02 (0.13)	0.01 (0.09)
Repair of humerus fracture	<0.01 (0.02)	<0.01 (0.04)	<0.01 (0.04)	0.01 (0.07)	0.01 (0.08)	0.01 (0.11)	0.01 (0.09)	0.01 (0.09)	0.01 (0.12)	0.01 (0.11)	0.01 (0.1)	0.02 (0.13)
Posterior spinal fusion for scoliosis	0.05 (0.21)	0.06 (0.23)	0.05 (0.21)	0.04 (0.2)	0.04 (0.19)	0.03 (0.17)	0.05 (0.22)	0.05 (0.21)	0.03 (0.18)	0.05 (0.22)	0.05 (0.22)	0.04 (0.2)
Appendectomy for acute appendicitis	0.03 (0.16)	0.03 (0.17)	0.02 (0.15)	0.03 (0.17)	0.03 (0.17)	0.02 (0.15)	0.03 (0.18)	0.04 (0.18)	0.03 (0.16)	0.04 (0.19)	0.04 (0.19)	0.03 (0.17)

Abbreviations: CH-A, children’s hospital tier A (freestanding children’s hospital); CH-B, children’s hospital tier B (children’s hospital attached to adult hospital); NCH, non–children's hospitals.

**Table 3.** Regression Coefficients for 90-Day Complications, 90-Day Readmissions, and Log Negotiated Hospital Payments by Commercial Insurers After Adjusting for Zip Code, Year, Month, Surgery, Surgery Setting, Complex Chronic Conditions, and Observable Patient, Hospital, and County Characteristics

Hospital type	Outcomes coefficients, 90 d^a^				Hospital payment coefficients^b^															
	Complications		Readmissions		All procedures				Appendectomy				Humerus fracture				Tonsillectomy			
	All inpatient procedures	*P* value	All inpatient procedures	*P* value	Inpatient	*P* value	Outpatient	*P* value	Inpatient	*P* value	Outpatient	*P* value	Inpatient	*P* value	Outpatient	*P* value	Inpatient	*P* value	Outpatient	*P* value
CH-A	0.23	.68	0.26	.56	0.39	<.001	0.34	<.001	0.43	<.001	0.33	.16	0.15	.61	0.27	.3	0.3	.12	0.46	<.001
CH-B	−0.05	.81	−0.02	.93	−0.02	.54	0.03	.42	−0.03	.41	0.11	.30	−0.06	.51	0.20	.16	−0.04	.63	0.02	.47
NCH	[Reference]		[Reference]		[Reference]		[Reference]		[Reference]		[Reference]		[Reference]		[Reference]		[Reference]		[Reference]	

Abbreviations: CH-A, children’s hospital tier A (freestanding children’s hospital); CH-B, children’s hospital tier B (children’s hospital attached to adult hospital); NCH, non–children's hospitals.

^a^ Regression coefficients are estimated using a generalized linear model with a binomial family and logit link function.

^b^ Regression coefficients are estimated using an ordinary least squares model.

### Negotiated Hospital Payments

After adjusting for zip code, year, month, surgery, surgery setting, complex chronic conditions, and observable patient, hospital, and county characteristics, we estimated payments for inpatient common procedures were 39% higher at CH-A than at NCH and 2% lower at CH-B than NCH. Payments for outpatient common procedures were 34% higher at CH-A than at NCH and 3% higher at CH-B than NCH. Inpatient and outpatient appendectomy, humerus fracture repair, and tonsillectomy payments were higher at CH-A than NCH. Inpatient appendectomy, humerus fracture repair, and tonsillectomy payments were lower at CH-B than NCH. Outpatient appendectomy, humerus fracture repair, and tonsillectomy payments were higher at CH-B than NCH (Table 3).

## Discussion

There is increasing desire for consumers to understand the value proposition for rising health care expenditures in terms of clinical outcomes and costs.^13^ Value-based purchasing strategies for employer health plans have been discussed for more than a decade but have been slow in adoption.^26,27^ The slow adoption surrounds poor definitions of value both in terms of outcomes and costs. Our study found that for commonly performed pediatric procedures, CH have comparable clinical outcomes but higher costs and thus lower value compared with NCH. To our knowledge, no prior studies have examined the value of CH for commonly performed procedures using payment data.

Prior studies attempting to assess value rely upon costs estimated using hospital-level charges rather than actual payments.^28,29^ Hospital charges are problematic because they rely upon inflated figures that typically far exceed negotiated payments. Furthermore, charge-to-cost conversion ratios are hospital-specific and preclude reliable hospital comparison. In contrast, we used payments from private insurance carriers, which are superior to charges and estimated costs because payments are a direct measure of prices paid for care. Payment data provide a better measure of the costs of care from a patient and societal perspective. Financial transparency is lacking and has been a barrier to this kind of work in the past. Using a novel approach made possible by access to the HCCI data set of hospital payments, our research is the first to examine actual payments across CH and NCH from 4 of the nation’s largest insurers.

Another challenge to assess value is that outcomes for common procedures in children are favorable with low rates of complications. Our study found there was no significant difference in the rate of surgical complications or 90-day readmissions at any of the hospital types. Complications for children undergoing surgery are typically associated with procedure complexity and patient comorbidities. Although some surgical procedures evaluated (eg, spine surgery) may have increased complexity (eg, severity of scoliosis), our results were consistent across the continuum of procedures studied. We attempted to limit procedural selection to the least complex procedural coding groups (eg, posterior spine approaches vs anterior approaches). Furthermore, we purposely sampled inpatient and outpatient procedures from a wide variety of children’s surgical subspecialities including general surgery, otolaryngology, orthopedic surgery, urology, ophthalmology, and neurosurgery. In pediatric populations, complications such as readmission are, in large part, associated with complex chronic conditions.^30^ Children undergoing the routine surgical procedures evaluated tend to be healthy. Our study adjusted for pediatric complex chronic conditions using the Feudtner classification system^22,23,24,25^ and still found higher payments to CH than NCH. Although CH typically care for a high proportion of patients with complex chronic conditions, accounting for patient complexity in our models as well as the selection of commonly performed surgical procedures attempts to ensure comparable assessment of CH and NCH.

It has been shown that clinical data are better than claims data when assessing complication rates; however, for the selected routine, commonly performed procedures with rare complication event rates, we expect claims data would be reliable. Additionally, we evaluated readmission rates, which are considered a more reliable outcome metric regardless of whether claims or clinical data are used.^31^

Variation exists in how hospitals are defined as CH in the pediatric literature. We used a previously described rigorous method to classify hospitals as either CH or NCH based on AHA survey results, publicly available data, and proportion of pediatric discharges based on HCCI data to validate the classifications.^21^ Multiple sensitivity analyses were performed to compare CH-A with CH-B and with NCH and so on, and we found consistent results.^21^

Finally, CH may receive higher payments than NCH on routine surgical procedures because of higher costs and lower reimbursements associated with pediatric populations. NCH may be able to spread care delivery costs across larger cohorts of patients, including adult populations who may have higher reimbursement rates.^32^ Furthermore, CH care for a disproportionate number of uninsured or publicly insured patients compared with NCH.^33,34^ Last, freestanding CH are typically smaller than NCH with fewer beds and lower overall volumes leading to higher equipment and supply costs. Although these trends may justify higher payments to CH for delivery of similar care as NCH, evaluation from the patient (consumer) perspective demonstrates lower individual value at CH.

### Limitations

This study has several limitations. First, HCCI data reflect payments and care delivery for employer-based and privately insured patient populations. Depending on the state, Medicaid (including Medical Assistance, Children's Health Insurance Plan [CHIP] or other government-assistance plan coverage) ranges from 17% (Utah) to 56% (New Mexico).^35^ Our results may be biased and thus have limited generalizability to publicly insured populations. Nevertheless, the large sample sizes and variable private insurance plans (with high and low deductible plans included) may increase the generalizability of our findings. Second, although these analyses clustered CH categories, we were unable to account for specific payments or outcomes based on nuances such as surgeon specialization. This may lead to unmeasured differences in patient or physician characteristics across the hospital-types assessed. Third, we did not evaluate referral practices and patient and/or family preferences toward undergoing surgical care at CH as compared with NCH. There may be a premium patients and families are willing to pay to undergo care at CH, assuming patients and families have freedom to select the definitive treating facilities and are not limited by insurer or policy factors. Last, using data from 2010 to 2015 may be criticized as dated. These analyses were conducted using the most contemporary data available at the initiation of this multiyear project and required extramural funding. As noted, payment data of this scope are rare, and this study represents one of the first of its type. With health care spending rising and pediatric care increasingly concentrated among specialized CH, our estimates may underestimate present-day payment differentials.

## Conclusions

For commonly performed surgical procedures in children, clinical outcomes are equivalent at CH and NCH but are associated with higher payments at CH, and thus lower overall value care. These results may not reflect all aspects of health care delivery that may define value for an individual patient and there may be a premium for which patients and families and insurers are willing to pay for access to CH. Nevertheless, with increasing focus on value-based care, further research is needed to evaluate mechanisms to decrease costs and improve value at both CH and NCH.

## Notes

Supplement. **eTable 1.** Procedures Included in the Analysis Were Identified by Current Procedural Terminology (CPT) and International Classification of Diseases, Ninth Revision (ICD-9) Codes **eTable 2.** Complications Used to Evaluate Post-Operative Outcomes Were Identified Using International Classification of Diseases, Ninth Revision (ICD-9) Codes
