# Supplementary material for: Association of Children’s Hospital Status With Value for Common Surgical Conditions
Source: JAMA Netw Open. 2022 Jun 24;5(6):e2218348. doi: 10.1001/jamanetworkopen.2022.18348 (PMC9233238; doi:10.1001/jamanetworkopen.2022.18348)
Supplement: Supplement. — eTable 1. Procedures Included in the Analysis Were Identified by Current Procedural Terminology (CPT) and International Classification of Diseases, Ninth Revision (ICD-9) Codes eTable 2. Complications Used to Evaluate Post-Operative Outcomes Were Identified Using International Classification of Diseases, Ninth Revision (ICD-9) Codes [file jamanetwopen-e2218348-s001.pdf]

Supplemental Online Content

Raval MV, Reiter AJ, McCarthy IM. Association of children's hospital status with value for common surgical conditions. *JAMA Netw Open.* 2022;5(6):e2218348. doi:10.1001/jamanetworkopen.2022.18348

eTable 1. Procedures Included in the Analysis Were Identified by Current Procedural Terminology (CPT) and International Classification of Diseases, Ninth Revision (ICD-9) Codes
eTable 2. Complications Used to Evaluate Post-Operative Outcomes Were Identified Using International Classification of Diseases, Ninth Revision (ICD-9) Codes

This supplemental material has been provided by the authors to give readers additional information about their work.

© 2022 Raval MV et al. *JAMA Network Open.*

eTable 1. Procedures Included in the Analysis Were Identified by Current Procedural Terminology (CPT) and International Classification of Diseases, Ninth Revision (ICD-9) Codes

Procedure	CPT Codes	ICD-9 Procedure Codes
Anterior Cruciate Ligament Reconstruction	27332, 27333, 29879, 29880, 29881, 29882, 29883, 29888	80.06, 80.16, 80.26, 80.36, 80.46, 80.6, 80.76, 80.86, 80.96, 81.22, 81.42, 81.43, 81.44, 81.46, 81.47
Anti-Reflux Surgery	43280, 43327, 43328	44.67, 44.66
Appendectomy	44950, 44960, 44970	47.0, 47.01, 47.09
Humerus Fracture Repair	24500, 24505, 24515, 24516, 24530, 24535, 24538, 24545, 24546, 24560, 24565, 24566, 24575, 24576, 24577, 24579, 24582	79.01, 79.11, 79.21, 79.31
Tympanostomy Tube Placement	69420, 69421, 69433, 69436	20.0, 20.01, 20.09
Tonsillectomy and Adenoidectomy	42820, 42821, 42825, 42826, 42830, 42831, 42835, 42836	28.2, 28.3, 28.6
Strabismus Surgery	67311, 67312, 67314, 67316, 67318	15.11, 15.12, 15.13, 15.2, 15.21, 15.22, 15.4
Posterior Spinal Fusion	22800, 22802, 22804	81.0, 81.00, 81.03, 81.05, 81.08
Cholecystectomy	47562, 47563, 47564	51.23
Umbilical Hernia Repair	49580, 49585	53.4, 54.41, 53.42, 53.49

© 2022 Raval MV et al. *JAMA Network Open.*

Inguinal Hernia Repair	49491, 49495, 49500, 49595, 49529, 49650	53.0, 53.1, 17.1, 17.2
Orchiopexy	54640, 54650, 54692	62.5
Circumcision	54150, 54160, 54161	64.0

eTable 2. Complications Used to Evaluate Post-Operative Outcomes Were Identified Using International Classification of Diseases, Ninth Revision (ICD-9) Codes
For brevity, the * represents all fourth or fifth digits that could designate an ICD-9 code. For example, 4151* = 41511, 41512, and 41519.

Complication	ICD-9 Code
Wound Complications	998.83, 998.12, 998.13, 998.3, 998.6
Superficial Surgical Site Infection	998.32, 998.5, 998.51, 998.59
Deep/Organ-Space Surgical Site Infection	998.31, 567.22, 998.59
Urinary Tract Infection	599.0, 996.64
Renal Insufficiency	584*, 586*
Pneumonia	480*, 481, 482*, 483*, 484, 485, 486, 487.0, 997.31
Respiratory Failure	518.5,518.82
Sepsis	038*, 785.52, 790.7, 995.91, 995.92, 998.0
Deep Vein Thrombosis	4534*, 453.9
Pulmonary Embolism	415,1, 415.19
Myocardial Infarction	410*0, 410*1
Cardiac Arrest	427.5
Intraoperative Complications	998.11 (bleeding), 998.2 (accidental puncture and laceration), 998.4(retained foreign body)

© 2022 Raval MV et al. *JAMA Network Open.*
